# Exploring the relationship between math anxiety and gender through implicit measurement

**DOI:** 10.3389/fnhum.2012.00279

**Published:** 2012-10-15

**Authors:** Orly Rubinsten, Noam Bialik, Yael Solar

**Affiliations:** Department of Learning Disabilities, Edmond J. Safra Brain Research Center for the Study of Learning Disabilities, University of HaifaHaifa, Israel

**Keywords:** arithmetic, anxiety, gender, effective priming, academic career

## Abstract

Math anxiety, defined as a negative affective response to mathematics, is suggested as a strong antecedent for the low visibility of women in the science and engineering workforce. However, the assumption of gender differences in math anxiety is still being studied and results are inconclusive, probably due to the use of explicit measures such as direct questionnaires. Thus, our primary objective was to investigate the effects of math anxiety on numerical processing in males and females by using a novel affective priming task as an indirect measure. Specifically, university students (23 males and 30 females) completed a priming task in which an arithmetic equation was preceded by one of four types of priming words (positive, neutral, negative, or related to mathematics). Participants were required to indicate whether the equation (simple math facts based on addition, subtraction, multiplication, or division) was true or false. People are typically found to respond to target stimuli more rapidly after presentation of an affectively related prime than after an affectively unrelated one. In the current study, shorter response latencies for positive as compared to negative affective primes were found in the male group. An affective priming effect was found in the female group as well, but with a reversed pattern. That is, significantly shorter response latencies were observed in the female group for negative as compared to positive targets. That is, for females, negative affective primes act as affectively related to simple arithmetic problems. In contrast, males associated positive affect with simple arithmetic. In addition, only females with lower or insignificant negative affect toward arithmetic study at faculties of mathematics and science. We discuss the advantages of examining pure anxiety factors with implicit measures which are free of response factors. In addition it is suggested that environmental factors may enhance the association between math achievements and math anxiety in females.

## Introduction

The nexus of educational policies, evidence based teaching practices, and cognitive neuroscience promises to use cutting-edge scientific methods and concepts to promote the growth and success of children. Mathematics is a focal point in this new synthesis because it is an important portal to knowledge in our western and technological age, from calculating change in shops to developing new computerized programs. As an example, intact numerical abilities are important for health numeracy (Nelson et al., 2008) and contribute to full-time employment in adulthood (Rivera-Batiz, 1992). This makes numerical cognition and disabilities an extremely important scientific field with potential application for solving problems in society and education.

Learning arithmetic or mathematics is, however, complicated for many people (Dowker, 2005) who have math anxiety, a persistent negative reaction to math (henceforth math anxiety), ranging from mild discomfort to extreme avoidance (Hembree, 1990; Ma and Xu, 2004a,b; Ashcraft and Ridley, 2005). Specifically, math anxiety may include feelings of tension (Richardson and Suinn, 1972), low self confidence in the ability to learn mathematics (Jain, 2009), and a decline in working memory (Ashcraft and Kirk, 2001), counting abilities (Maloney et al., 2010) and the precision of the mental representations of numerical magnitudes (Maloney et al., 2012).

Ashcraft et al. (2007) suggested that if a statistical cut-off for high math anxiety is maintained at one standard deviation above the mean, roughly 17% of the population would be labeled as high math anxious. Such a definition suggests that nearly one fifth of the population experiences high math anxiety. The incidence of mild math anxiety among college students is estimated at around 50% (Betz, 1978). Specifically, Betz found that approximately half the college students in their sample (652 college students from either math or psychology courses) indicated that math made them feel “uncomfortable, nervous, uneasy, and confused.” Expressions of anxiety were most common when items concerned math tests; about half the students in all three groups reported being “really uptight” during math tests.

Though there have been exceptions, most studies of math anxiety have reported higher levels of math anxiety for females than for males (e.g., Betz, 1978; Wigfield and Meece, 1988; Hembree, 1990; Bernstein et al., 1992; Ashcraft and Faust, 1994; Hopko, 2003; Hopko et al., 2003; Ma and Cartwright, 2003; Haynes et al., 2004; Woodart, 2004; Baloglu and Kocak, 2006; McGraw et al., 2006; Jain, 2009; Else-Quest et al., 2010). However, other studies failed to find such a gender difference (e.g., Hackett, 1985; Cooper and Robinson, 1991). Even mild levels of math anxiety have been shown to be associated with later academic decisions (Brown et al., 2010). Moreover, Hackett (1985) found that perceptions of mathematics self-efficacy were the most powerful predictors of occupational choice. This may suggest that math anxiety may be a strong antecedent for the low visibility of women in the science and engineering workforce. For example, despite gender similarities in math achievements (Hedges and Nowell, 1995; Hyde et al., 2008; Else-Quest et al., 2010) or even better math grades for females compared to males (Kenney-Benson et al., 2006), women make up only 24% of the science and engineering workforce (National Science Foundation., 2002). Moreover, Long (2001) found that women are less likely to be full professors, tenured, or hold tenure track science and engineering academic positions. Indeed, it has been suggested that, as our society becomes increasingly dependent on numbers and math, failure to acquire numerical skills may come to act as a “critical filter,” limiting occupational success for women (e.g., Halpern et al., 2007).

However, as mentioned earlier, the question of gender differences in math anxiety is still under huge debate (e.g., Betz, 1978; vs. Cooper and Robinson, 1991; or Hackett, 1985) despite its significance in education and daily life. One variable that might be related to different findings regarding gender differences in math anxiety is the common use of explicit tools such as the math anxiety rating scale (e.g., Richardson and Suinn, 1972), the math anxiety questionnaire (Wigfield and Meece, 1988), (for a German version see: Krinzinger et al., 2007) or the revised Math Anxiety Rating Scale (MARS-R: Alexander and Martray, 1989; Hopko, 2003) to diagnose math anxiety. Such explicit tools typically assess accessible self-representations.

Explicit questionnaires have been the primary method for obtaining information on symptoms of math anxiety and other psychopathology in the school setting, in part because of convenience, standardization, and good psychometric properties (as suggested for example in cases of ADHD—Pelham et al., 2005). However, women have consistently been found to score higher than men on self-report measures of trait anxiety (e.g., Feingold, 1994; Costa et al., 2001; Egloff and Schmukle, 2004), possibly resulting from gender differences in anxiety that are not due to anxiety *per se*. Indeed, Flessati and Jamieson (1991) argued that the gender difference in math anxiety could be explained by the fact that females are more self-critical of math anxiety and of their performance in mathematics.

Implicit measures, on the other hand, typically assess inaccessible cognitive structures or presentations that are being processed automatically. It has been shown that affective traits can be activated automatically and influence emotional, cognitive, or behavioral processes (e.g., Giner-Sorolla et al., 1999). That is, affective processing begins immediately and even involuntarily upon seeing a salient affective word or picture. Psychologists use situations where implicit processing is possible in order to study automaticity. One such task, used in the current work, is the priming task, in which an early stimulus (i.e., prime) designed to be ignored influences the response to a subsequent relevant stimulus. In many cases, participants cannot ignore the irrelevant dimension (the prime), which facilitates or interferes with the processing the relevant one (the target).

Egloff and Schmukle (2004) found that the effect sizes of the gender differences in implicit anxiety measures were approximately half as large as the ones in the explicit tests. Such findings suggest that indeed explicit anxiety measures may be influenced (although not exclusively) by biased self-reports. That is, differences between implicit and explicit measures have emerged because implicit measures may be free of response factors. Hence, the finding that there were still gender differences in implicit anxiety measures is probably due to pure anxiety factors that lead to gender differences. Accordingly, implicit anxiety measures and more specifically, implicit math anxiety measures may be the way to go when studding gender differences. That is, our main focus is to use the idea of an implicit measure, as a good tool to study anxiety and gender (Egloff and Schmukle, 2004), in order to study math anxiety (which has not been studied so far).

Therefore, here, and based on previous work (Rubinsten and Tannock, 2010), we use a novel arithmetic-affective priming task to study gender differences in math anxiety. Affective priming studies have demonstrated that people respond to target stimuli more quickly after presentation of an affectively related prime stimulus than after one that is affectively unrelated, whether the target involves written words or not (e.g., naming target' written words: Hermans et al., 1994; Bargh et al., 1996; Cassotti et al., in press; naming or categorizing pictures: Spruyt et al., 2004) (for review see De Houwer et al., 2009).

Rubinsten and Tannock (2010) developed a novel arithmetic-affective priming task with four different types of primes (words with positive, neutral, and negative affect, as well as words related to mathematics such as “multiplication” or “quantity”) with single-digit arithmetic problems (such as 3 + 4 = 7) as targets. Participants were simply required to decide if the target was true or false. The authors used this novel arithmetic-affective priming task to study math anxiety, focusing on how the presentation of affective word-primes influences the ability to solve simple arithmetic problems. It was found that affective priming indeed shows math anxiety. Specifically, there was a significant difference between developmental dyscalculic (DD) children, who typically have a basic deficit in math skills and struggle with even the simplest math tasks (for review see Rubinsten and Henik, 2009), and control children in their reaction time (RT) to positive, negative, and math related prime words while judging simple math equations to be true or false (Rubinsten and Tannock, 2010). The DDs reacted significantly faster when the equations were preceded by negative and math related primes, thus associating math with negative affect. The controls, on the other hand, reacted faster when the primes were positive and reacted to math related primes the same way as they reacted to neutral ones, showing that math has a neutral valence for them. In other words, participants in the DD group do not like arithmetic compare to controls (who do like arithmetic), and in terms of the definition of math anxiety this means that DDs showed more math anxiety (for more details about the exact method see Rubinsten and Tannock, 2010).

In the current study, we used Rubinsten and Tannock's affective-priming tool on typically developing male and female university students, who have been dealing with math for a considerable amount of their lives and whose math concepts and anxieties are probably well established. We use this novel arithmetic-affective priming task to study math anxiety and gender differences, focusing on how the presentation of affective word-primes such as “war” or “love” influences the ability to solve simple arithmetic facts (e.g., 5 + 2 = 7 or 3 × 4 = 12). Royera et al. (1999) found that males, at a broad range of ages including college students, are quicker than females at retrieving simple arithmetic facts. What could lead to such a gender difference in solving simple arithmetic? Could it be math anxiety that might have an impact on some of the cognitive functions involved with solving simple arithmetic facts? Specifically, with increasing practice or skill, children and adults were shown to automatically retrieve the solutions to very simple addition (e.g., 3 + 4 = 7: Barrouillet and Lépine, 2005; Thevenot et al., 2007) and multiplication math problems (e.g., 3 × 4 = 12: Ischebeck et al., 2007; Grabner et al., 2009) from verbal memory as the strategy of choice without involving quantity processing (although see Campbell and Alberts, 2009) (e.g., Siegler, 1987). In contrast, single-digit subtraction and, sometimes, simple division appear to activate a distinct neural network (Kong et al., 2005), suggesting that subtraction and division (and maybe even addition; Venkatraman et al., 2005) require manipulation of very basic representations of mental numerical magnitudes and quantities (Dehaene et al., 2003; Lemer et al., 2003; Takayama et al., 1994; Dehaene et al., 1999). Recently Rosenberg-Lee et al. (2011) argued that strategies used to solve division problems are much more complex than strategies used to solve subtraction problems and include processing of inverse relations which are operation specific.

Math anxiety has been shown to influence these cognitive functions (e.g., magnitude or quantity manipulations etc.; Maloney et al., 2010, 2012) known to be involved in simple arithmetic and hence, we investigated if gender differences in math anxiety indeed have a direct influence on solving simple arithmetic facts, resulting in gender differences in arithmetic (Royera et al., 1999). Specifically, Maloney et al. (2010, 2012) have shown that math anxiety has an influence on very basic numerical abilities such as counting and mental representations of numerical magnitudes. However, Ashcraft and colleagues (Ashcraft and Faust, 1994; Faust et al., 1996) found that math anxiety affects only complex arithmetic problems (e.g., arithmetic with carrying), and had little effect on simple addition and multiplication problems. Accordingly, we ask here (1) whether a gender difference in the ability to solve arithmetic facts (Royera et al., 1999) could be due to math anxiety, (2) whether math anxiety influences basic numerical abilities (Maloney et al., 2010, 2012) needed to solve simple arithmetic facts, and (3) whether the affective priming tool can be a good measure for studying such a difference (i.e., can we replicate the findings of Rubinsten and Tannock, 2010).

We predicted that (1) A direct link would appear between emotions (primes) and arithmetic problem solving (targets); (2) Compared to males, females would respond quicker to targets (i.e., simple arithmetic facts) preceded by negative affective primes that, in this group, would act as affectively related primes; and (3) affective differences will have a significant influence on career decisions. That is, we assumed that only those females who have less or insignificant negative affect toward arithmetic, study in the faculty of mathematics and sciences (vs. females who study in the faculty of humanities). (4) Math primes (e.g., words like “quantity”) would have the same effect as negative affective primes (i.e., act as affectively related primes) in the female group but not in the controls, at least for some of the arithmetic problems (i.e., targets).

## Methods

### Participants

A total of 56 students from the University of Haifa participated in the experiment. Of these, 23 were males (mean age 26.93 years, SD 6.90) and 33 females (mean age 26.24 years, SD 3.45). The male and female groups were matched for age, arithmetic abilities, attentional abilities, and reading abilities. Accordingly, five female participants (mean age 27.30 years; SD 3.27) were excluded from the experiment as they didn't meet inclusion/exclusion criteria, leaving a total of 51 participants (see inclusion/exclusion criteria below for more details). All participants in the final sample were either from the humanities faculty (15 males and 18 females) or from sciences, engineering, and math (8 males and 10 females).

Both groups of participants were recruited through advertisements distributed on the campus of the University of Haifa. Participants gave written consent to participate in the experiment and were paid about 20 shekels for their participation. Recruitment, payment, tasks, and overall procedure of the current specific study were authorized by the Research Ethics Committee of the University of Haifa (approval number 108/09). Written informed consent were obtained from all participants involved the current study.

#### General inclusion/exclusion criteria

All participants had no sensory or physical impairments (which would preclude participation in the computerized or paper-and-pencil testing), and no current or previous history of psychosis or other mental health disorders (e.g., ADHD, anxiety, or depression) that might influence cognitive performance. These details (and other details concerning medical and learning history) were confirmed by a personal information form the participants were required to complete.

***Classification measures***. To participate in the experiments, participants had to meet the following criteria. Male and female groups were matched for classification measures results, as can be seen in Table 1:

1. No previous or current diagnosis of ADHD, as reported by the participants. According to the Diagnostic and Statistical Manual of Mental Disorders: DSM-IV, symptoms of ADHD (attention or hyperactivity/impulsivity) must appear before the age of seven (American Psychiatric Association, 1994). Therefore, participants were only excluded from the analysis if they reported a childhood history of ADHD and also reported six out of nine symptoms relating to inattention or hyperactivity/impulsivity on a questionnaire of current difficulties in the attention domain, based on the DSM-IV (American Psychiatric Association, 1994).
2. No learning problem specific to the domain of reading, as reported by participants and shown by scores in the normal range (standardized scores between −2 to 2) on Rapid Automatized Naming (RAN) of letters and single-word decoding task. In the RAN test, participants were required to name the Hebrew letters they saw as fast as they can. In the word decoding task, participants were required to read the Hebrew words they saw as quickly and accurately as they could.
3. No impaired numeracy skills as reported by participants in an arithmetic difficulties questionnaire and shown by scores of normal range (standardized scores between −3 and 3) on a 2 min arithmetic problem solving task (Oppenheim-Bitton and Breznitz, 2003). In this test, participants were required to solve simple arithmetic problems as accurately and as quickly as they could, with a maximum time of 2 min (see Table A1 for the test itself).

**Table 1:** **Gender differences based on classification measures**.

	**Single word decoding task–accuracy (correct responses)**	**Single word decoding task–speed (time in seconds)**	**RAN–speed (time in seconds)**	**Arithmetic two-minute task–accuracy (number of errors)**	**Arithmetic two-minute task–number of problems solved**
**MALES**					
*n*	23	23	23	18	18
Mean	99.13	57.79	21.35	1.00	77.50
Standard deviation	0.73	9.33	2.76	1.50	6.58
**FEMALES**					
*n*	28	28	28	23	23
Mean	99.00	60.73	21.94	0.91	74.00
Standard deviation	0.79	8.64	3.16	0.85	9.86
***T* TEST**					
	*t*_(49)_ = −0.61, *p* > 0.05	*t*_(49)_ = 1.17, *p* > 0.05	*t*_(49)_ = 0.70, *p* > 0.05	*t*_(39)_ = −0.24, *p* > 0.05	*t*_(39)_ = −1.31, *p* > 0.05

### Arithmetic-affective priming task

#### Stimuli

Each trial consisted of a prime (one of the four types of affective words) and target (simple arithmetic equation) that appeared sequentially. Both prime and target appeared horizontally at the center of a computer screen in black characters against a white background. Each character was printed in boldface in Ariel font, size18, with a visual angle of 1.5°.

***Primes***. A list of 40 words (10 negative, 10 positive, 10 neutral, and 10 mathematics words) comprised the primes (see Table A2 for details of the primes). Valences for the emotional and neutral words were taken from the study by Besser et al. (2008). Most extreme negative and positive affective words were selected. Among those extreme words primes were also selected according to their length and part of speech to create similar characteristics. Note that there are no norms for emotional values of mathematical words.

***Targets***. Equations were presented in the form “a × b = c,” where *a* and *b* represented single digits from 1 to 9, × represented an arithmetic operation (×, +, −, or ÷), and *c* represented the solution. For *a* and *b* we employed all possible pairs of digits from 1 to 9 such that (1) regardless of the arithmetic operation used, the solution to the equation was a positive integer; and (2) the four arithmetic operations produced different solutions. For example, 7 × 3 and 5 × 4 were excluded because in these cases, division results in a solution that is not an integer. Likewise, equations such as 3 × 1 were excluded, where multiplication and division produce the same result, and 4 × 2, where the same is true for division and subtraction. Five pairs of digits meet both criteria (9 × 3, 8 × 4, 8 × 2, 6 × 3, and 6 × 2), and so these pairs were included in the experimental blocks. For all stimuli, the numerically larger digit was presented on the left side.

There were two types of solution conditions as follows: (1) The true condition comprised 20 equations with true results according to the criteria described above (e.g., 8 × 4 = 32); (2) For each of the 20 true equations there was one false equation by borrowing the solution to another equation (as long as this solution was not the same as *a* or *b*, i.e., a digit belonging to the arithmetic fact itself). For example, for the stimulus 8 × 4, the false solution was 3, which is the true result for 6 ÷ 2, (See Table A3 for the target arithmetic problems).

Each participant underwent 160 trials using the 40 primes four times each, twice with a true equation as the target and twice with a false equation. For the true condition, each prime appeared with two different true equations that were pseudo-randomly selected such that any given true equation appeared only once for each group of 10 primes. This produced a total of 80 true equations. For the false condition, each prime appeared with two different false equations, which once again were pseudo-randomly selected, such that any given false equation appeared once in each group of 10 primes. This produced a total of 80 false equations. The following two variables were included in the analysis: group (males vs. females), prime (negative, positive, neutral, and math), and target's arithmetic operation (+, −, ×, ÷). Thus, we had a 2 × 4 × 4 factorial design. Group was the only between-participants variable and primes and targets were manipulated within block.

Before the experiment began, participants completed a practice phase with eight primes and eight equations, four true, and four false (see Table A4 for description of primes and targets used in the practice session). The primes and equations were different than those used in the experiment itself.

### Procedure

Stimuli were presented on a computer screen at a distance of approximately 60 cm from participants. Participants were told that they were about to participate in a simple arithmetic experiment and that a word and simple arithmetic problem would be sequentially presented on the computer screen. They were instructed to decide if the arithmetic problem was correct or not as quickly as possible while ignoring the word. Reponses were made by pressing one of two possible keys.

Each trial opened with a 500 ms presentation of a fixation cross in the center of the computer screen. Five hundred milliseconds after offset of the fixation cross, the prime words were presented for 250 ms. The target arithmetic equation followed an offset of the prime words, resulting in a stimulus onset asynchrony (SOA) of 250 ms. The target equations were displayed until the participant responded “correct” or “incorrect” by pressing one of two keys on the keyboard (the letters “p” or “q”), or until 3000 ms had elapsed. The correct solution was represented by the letter “q” and the incorrect solution was presented by the letter “p.” The next trial was initiated 2000 ms after the participant's response. The computer measured RT in milliseconds from stimulus onset to participant's response.

## Results

Incorrect responses (3% in all) were discarded from the analysis. An accuracy analysis demonstrated that the number of errors made by the male group did not differ significantly from those made by the female group [*t*_(49)_ = −0.342, *p* > 0.05]. Also, the correlation between error rates and RT was insignificant (*r* = −0.221, *p* > 0.05), thereby excluding any speed-accuracy trade-off.

A Three-Way repeated measures ANOVA was used, including type of prime (i.e., affective valence: math, negative, neutral, and positive) and arithmetic procedure (addition, division, multiplication, and subtraction) as within-group variables and group (males and females) as the only between-group variable. Because Mauchly's Test of Sphericity indicated that circularity could not be assumed, all of the following *F*-statistics are adjusted by the Greenhouse-Geisser correction.

The main effect of arithmetic operation [i.e., target; addition: *M* = 997.23 ms, SD = 27.68 ms; division: *M* = 952.22 ms, SD = 26.5 ms; multiplication: *M* = 1000.23 ms, SD = 29.1 ms; subtraction: *M* = 955.87 ms, SD = 26.29 ms; *F*_(3, 147)_ = 11.32, *p* < 0.001], reached significance.

Of primary relevance to the aims of the current study was the significant interaction between group and type of prime, *F*_(3, 147)_ = 10.28, *p* < 0.001, showing that the difference between negative and positive primes is significantly different between males (negative minus positive: +59 ms) and females (negative minus positive: −38 ms), *F*_(1, 49)_ = 19.84, *p* < 0.001.

**Figure 1:**
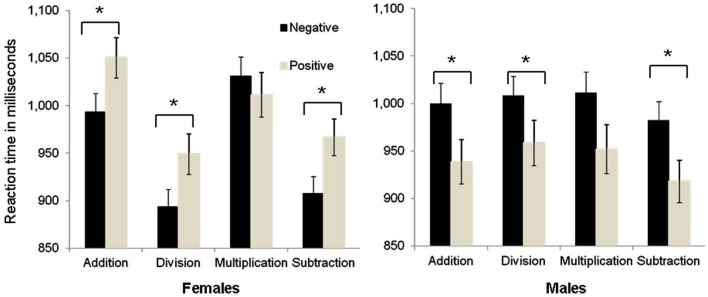
**Triple interaction between Prime (negative vs. positive), Arithmetic Procedure and Group.** Planned comparisons revealed an affective priming effect (i.e., positive affective primes vs. negative affective primes) in both groups but with a different pattern. Error bars denote the standard error of the mean. ^*^ = (*p* < 0.05).

Indeed, planned comparisons (see Figure 1) based on our initial hypothesis confirmed that a differential priming (relatedness) effect between the two groups was obtained in the priming data. Specifically, analysis revealed an affective priming effect (i.e., positive affective primes vs. negative affective primes) in both groups but with a different pattern. That is, significantly shorter response latencies for positive as compared to negative affective primes were found in the **male** group (i.e., positive affective primes tend to act as affectively related to simple arithmetic problems) when targets were *addition* problems (−61 ms difference between positive minus negative primes), *F*_(1, 22)_ = 8.27, *p* < 0.05; *division* (−50 ms difference between positive minus negative primes), *F*_(1, 22)_ = 4.86; *p* < 0.05; or *subtraction* (+61 ms difference), *F*_(1, 22)_ = 6.7, *p* < 0.05; and only marginal significance in *multiplication* problems (−61 ms difference between positive minus negative primes), *F*_(1, 22)_ = 3.3; *p* = 0.08.

An affective priming effect was found in the **female** group as well, but with a reversed pattern. That is, significantly shorter response latencies were observed in the female group for negative as compared to positive targets (i.e., *negative* affective primes act as affectively related to simple arithmetic problems) when targets were *addition* problems (+57 ms difference between positive minus negative primes), *F*_(1, 27)_ = 12.3, *p* < 0.01; *subtraction* (+59 ms difference between positive minus negative primes), *F*_(1, 27)_ = 7.1, *p* < 0.05; or *division* problems (+56 ms difference), *F*_(1, 27)_ = 4.8, *p* < 0.05; and an insignificant difference in multiplication problems. Accordingly, the affective priming effect in the female group (i.e., related\negative primes vs. unrelated\positive primes) is different from that found in the male group (i.e., related\positive primes vs. unrelated\negative primes).

To note, math and neutral related primes did not reach significance in both the female (*p* = 0.2 and *p* = 0.4 respectively) and the male (*p* = 0.4 and *p* = 0.5 respectively) groups.

We also wished to investigate if the significant affective priming effects (positive vs. negative primes) are similar within each faculty (i.e., faculty of humanities vs. faculty of mathematics and sciences). Accordingly, a Four-Way repeated measures ANOVA was used, including type of prime (i.e., affective valence: negative and positive) and arithmetic procedure (addition, division, multiplication, and subtraction) as within-group variables and group (males and females) and faculty (humanities vs. mathematics and sciences) as between-group variables. *F*-statistics are adjusted by the Greenhouse-Geisser correction. In both groups of faculty the interaction between prime (negative vs. positive) and gender was found to be significant; *humanities F*_(1, 33)_ = 12.2, *p* < 0.01; *mathematics and sciences F*_(1, 16)_ = 9.94, *p* < 0.01.

Interestingly however, and as can be seen in Table 2, arithmetic performance of female students in the *faculty of humanities* is significantly different from males' arithmetic performance. That is, the affective priming effect in the female group (i.e., related\negative primes vs. unrelated\positive primes) is different from that found in the male group (i.e., related\positive primes vs. unrelated\negative primes). It should be noticed that effect sizes of both these groups are quite similar. However, in the *faculty of mathematics and sciences*, despite the fact that *n* is similar in both groups and even a bit larger in the female group (females: *n* = 10; males *n* = 8), the effect size of the related\positive primes vs. unrelated\negative primes in the male group was very large (η^2^ = 0.75) and the effect was significant whereas in the female group the effect of related\negative primes vs. unrelated\positive primes' was not significant (and only η^2^ = 0.18).

**Table 2:** **Gender and faculty differences in negative vs. positive primes**.

	***n***	***Positive minus negative in ms***	***F***	**η^2^**
**FACULTY OF HUMANITIES**				
Females	18	+44	*F*_(1, 19)_ = 4.5, *p* < 0.05	0.22
Males	15	−59	*F*_(1, 14)_ = 6.0, *p* < 0.05	0.3
**FACULTY OF MATHEMATICS AND SCIENCES**				
Females	10	+35	*ns*	0.18
Males	8	−38	*F*_(1, 17)_ = 20.7, *p* < 0.01	0.75

## Discussion

The present study measured implicit valence of mathematics and results demonstrate that negative affect during simple arithmetic exists in females but not in males (i.e., in the case of females, *negative* affective primes act as affectively related to simple arithmetic problems). On the contrary, males typically associate positive affect with simple arithmetic (i.e., *positive* affective primes act as affectively related to simple arithmetic problems). That is, the results indicate that women like mathematics significantly less than men do and that they (women) relate negatively to it. Accordingly, and in terms of the accepted definition of math anxiety, the results may be interpreted as an expression of math anxiety in females. Interestingly, these affective differences have a significant influence on career decisions. That is, only those females who have less or insignificant negative affect toward arithmetic, study in the faculty of mathematics and sciences. To the best of our knowledge, this is the first study to demonstrate gender differences in math anxiety by using an implicit measure. Hence, the current study had the advantage of examining pure anxiety factors which are not biased upon self-report and are free of response factors (Egloff and Schmukle, 2004).

Notably, the exposure to math words did not result in any important significant results. To the best of our knowledge, besides Rubinsten and Tannock's (2010) study, there has been no research that used affective priming task with such primes or similar to them. Typically, studies use only positive and negative emotional primes and report on affective priming as the difference between RT to positive vs. negative primes. The use of math words in the current study (similarly to Rubinsten and Tannock's study) was a new attempt to use these words and to see if they might have any valence, but they didn't. However, the strong significant differences between positive and negative primes, as mentioned throughout the “Result” and “Discussion” below support our main hypothesis and clearly show the gender differences in the expression of math anxiety.

Why didn't math primes have a similar effect as negative primes for females, comparable to what was found in Rubinsten and Tannock, 2010? Rubinsten and Tannock's work included English math words which were mostly verbs. In the current work, which was administered in Hebrew, we used similar words but as adjectives and not verbs. We used adjectives since we had to keep length, frequency and other variables similar to the other types of primes. In addition and very importantly, in Hebrew, verbs are conjugated according the gender, number and person of the subject. Hence, the use of verbs would mean the need to use either the feminine or masculine conjugation which might have a huge influence on results (since the main objective here is to study gender and math). It could be that math primes as verbs, have a larger affective influence than adjectives since the person feels that he/she has to do (verb; e.g., the person feels that he actively dividing) math and affectively relate to the math word. Accordingly, this could be the reason why the study conducted in English (Rubinsten and Tannock; verbs), but not in Hebrew (adjectives), resulted in priming effects with the math primes. Further research, of course, is needed in this subject matter.

### Why do females have negative feelings about math?

Our results show that math anxiety (i.e., negative affect associated with math) is not similar among males and females, particularly in arithmetic that requires calculations using quantity manipulation (e.g., division, subtraction, and addition) (Kong et al., 2005; e.g., Siegler, 1987). On the other hand, math anxiety does not exist in females where there is need for verbal memory retrieval (e.g., multiplication 3 × 4 = 12: Ischebeck et al., 2007; Grabner et al., 2009). While these data do not allow us to state unequivocally why or how math anxiety develops and evolves, convergent with other reports of environmental and age-related math anxiety (Wigfield and Meece, 1988; Beilock et al., 2010), the current findings may suggest that females undergo significant age or environment-related changes that lead to math anxiety (i.e., negative affect associated with math) during the processing of simple arithmetic. That is, math anxiety in females could be the result of biological and developmental factors that interfere with intact development of basic numerical abilities such as quantity manipulation (which may result, as we found, in difficulties with solving simple arithmetic involving subtraction, addition, and division). Such an argument is consistent with previous studies (Maloney et al., 2010, 2012) indicating that math anxiety may result from a basic low-level deficit in numerical processing that compromises the development of higher level mathematical skills and, as indicated by the current data, have an influence on adults' career decisions. That is, initial poor basic numerical abilities may precede and give rise to math anxiety, creating a vicious cycle in females but not in males. However, to the best of our knowledge, no study has reported gender differences in such basic numerical abilities (e.g., quantity processing) that have a biological base to date. Accordingly, it would make more sense to argue that environmental factors might affect females' attitude toward math. For example, Beilock et al. (2010) found a strong link between teachers with math anxiety and the math anxiety of their female students. Specifically, the more anxious teachers were about math, the more likely were their female (but not male) students to adopt the stereotype whereby “boys are good at math, and girls are good at reading.” In addition, Ma and Xu (2004a), suggested that math anxiety springs from the unpleasant memory of poor mathematics performance in the past. Accordingly, and together with the current findings, it seems reasonable to assume that most of the evaluative reactions toward math in females are learned rather than innate.

### Implicit vs. explicit measures of math anxiety

Contrary to previously used explicit measures, the affective priming task reveals pure differences in math anxiety between (1) gender and (2) the four arithmetic procedures. In terms of arithmetic procedures, the current findings, together with previous data (Rubinsten and Tannock, 2010), strongly challenge one of the most leading viewpoints on mathematics anxiety. Specifically, it had been argued that people who suffer from math anxiety only have difficulties with complex mathematics and not with simple arithmetic (Ashcraft and Faust, 1994; Faust et al., 1996). Here we show that the effect of math anxiety does in fact extend to simple arithmetic. It is possible that, contrary to the current study, previous studies (e.g., Ashcraft and Faust, 1994; Faust et al., 1996), which analyzed the four arithmetic procedures (multiplication, addition, division, and subtraction) as one, failed to find a difference in simple arithmetic due to strategy differences in those four procedures (e.g., verbal retrieval vs. quantity manipulations—see for example Kong et al., 2005; Ischebeck et al., 2007; Grabner et al., 2009). It is also possible that, contrary to previous work, the current implicit measure, i.e., the affective priming task, which is not biased by self-report, could be attributed to math anxiety *per se* and specifically to solving simple arithmetic problems.

As previously shown (e.g., Hembree, 1990), math anxiety manifests itself as an unpleasant emotional response to math. This is what we show here—simple arithmetic and math or negative words are implicitly associated with unpleasant emotions. Accordingly, it may be suggested that our arithmetic-affective priming may be used as an indirect measure of math anxiety.

In terms of gender differences, and also based on previous arguments (Greenwald et al., 2002), the current arithmetic affective priming task indicates that both males and females process information about their feelings in an implicit (i.e., automatic or unconscious) mode. Greenwald et al. (2002) have argued that respondents are incapable of reporting some implicit cognitive and affective processes because they operate outside their subjective awareness. Consequently, the current implicit affective priming task (unlike self-report) is able to measure such implicit emotional constructs. By using this task the construct of interest, i.e., math anxiety, is indirectly measured, thus bypassing the problems of introspective limits and response factors.

## Conclusions

The current findings are quite significant in terms of the relationship between math anxiety and gender. Environmental factors such as teachers' attitudes toward math (Beilock et al., 2010) seem to intensify the association between math achievements and math anxiety among females, further impeding arithmetic achievements and influencing career decisions. As math anxiety may be a strong antecedent for the low visibility of women in the science and engineering workforce (National Science Foundation., 2002), interventions that reduce negative attitudes toward math (e.g., Hendel and Davis, 1978; Tooke and Lindstrom, 1998; Gresham, 2007) in females from early childhood should become an important part of the math educational system.

### Conflict of interest statement

The authors declare that the research was conducted in the absence of any commercial or financial relationships that could be construed as a potential conflict of interest.

## Appendix

**Table A1:** **Arithmetic two--minute test (Oppenheim-Bitton and Breznitz, 2003).** Instructions: The following pages contain arithmetic problems. You have 2 min to solve as many equations as you can. Solve according to columns (A first, then B, then C, and D), and do not skip any of the equations.

**A**	**B**	**C**	**D**
2 + 2 =	3 + 5 =	5 + 6 =	7 + 5 =
4 − 2 =	6 − 4 =	8 − 3 =	10 − 3 =
2 × 3 =	3 × 5 =	6 × 7 =	8 × 4 =
4 : 2 =	9 : 3 =	32 : 8 =	27 : 3 =
2 + 3 =	3 + 6 =	6 + 6 =	8 + 9 =
5 − 2 =	7 − 4 =	9 − 8 =	10 − 2 =
2 × 4 =	3 × 6 =	6 × 8 =	8 × 9 =
6 : 2 =	12 : 3 =	36 : 6 =	72 : 9 =
2 + 4 =	4 + 4 =	6 + 7 =	9 + 6 =
5 − 4 =	7 − 5 =	9 − 5 =	10 − 6 =
2 × 5 =	4 × 5 =	6 × 9 =	9 × 7 =
8 : 2 =	12 : 6 =	42 : 7 =	48 : 6 =
2 + 5 =	4 + 5 =	6 + 8 =	9 + 5 =
5 − 3 =	8 − 5 =	9 − 7 =	10 − 5 =
3 × 3 =	5 × 6 =	7 × 4 =	9 × 9 =
10 : 2 =	15 : 5 =	18 : 3 =	42 : 7 =
3 + 4 =	5 + 5 =	7 + 4 =	9 + 7 =
6 − 2 =	8 − 6 =	9 − 6 =	10 − 7 =
3 × 4 =	6 × 6 =	7 × 7 =	8 × 8 =
6 : 3 =	24 : 4 =	64 : 8 =	21 : 3 =

Number of total answers:

Number of correct answers:

Number of incorrect answers:

Time (if all equations were solved before 2 min had passed):

**Table A2:** **Primes used in the experiment—list and description**.

**Negative words**			**Positive words**		
**Word**	**Length of Hebrew word**	**Part of speech in Hebrew**	**Word**	**Length of Hebrew word**	**Part of speech in Hebrew**
Shortage	5	Noun	Achievement	4	Noun
Failure	5	Noun	Success	5	Noun
Withdrawal	5	Noun	Summit	4	Noun
Defeat	5	Noun	Promotion	5	Noun
Delay	5	Noun	Status	4	Noun
Rejection	5	Noun	Sharing	5	Noun
Detachedness	5	Noun	Appreciation	5	Noun
Insulting	5	Noun	Friendship	5	Noun
Confrontation	5	Noun	Giving	5	Noun
Separation	5	Noun	Support	5	Noun
**Math-related words**			**Neutral words**		
Math	7	Noun	Notebook	5	Noun
Addition	5	Noun	Sharpener	4	Noun
Number	4	Noun	Printer	5	Noun
Count	5	Noun	Envelope	5	Noun
Division	5	Noun	Binder	4	Noun
Subtraction	5	Noun	Fluid	5	Noun
Multiplication	3	Noun	Telephone	5	Noun
Minus	5	Noun	Staple	4	Noun
Quantity	4	Noun	Document	4	Noun
Plus	4	Noun	Perforator	5	Noun

**Table A3:** **Targets used in the experiment**.

**Targets appearing in experiment trials**	
**Incorrect equations**	**Correct equations**
6 − 2 = 9	6 − 2 = 4
6 − 3 = 32	6 − 3 = 3
6 : 2 = 18	6 : 2 = 3
6 : 3 = 16	6 : 3 = 2
6 + 2 = 16	6 + 2 = 8
6 + 3 = 8	6 + 3 = 9
6 × 2 = 9	6 × 2 = 12
6 × 3 = 4	6 × 3 = 18
8 − 2 = 3	8 − 2 = 6
8 − 4 = 3	8 − 4 = 4
8 : 2 = 9	8 : 2 = 4
8 : 4 = 6	8 : 4 = 2
8 + 2 = 16	8 + 2 = 10
8 × 2 = 27	8 + 4 = 12
8 + 4 = 6	8 × 2 = 16
8 × 4 = 3	8 × 4 = 32
9 : 3 = 10	9 − 3 = 6
9 + 3 = 16	9 : 3 = 3
9 + 3 = 2	9 + 3 = 12
9 × 3 = 8	9 × 3 = 27

**Table A4:** **Primes and targets used in the practice phase**.

**Primes appearing in practice trials**			
**Positive words**	**Negative words**	**Neutral words**	**Math-related words**
Prosperous	Deficit	Stapler	Arithmetic
Affection	Argument	Ruler	Ten
**Targets appearing in practice trials**			
**Incorrect equations**		**Correct equations**	
3 + 1 = 5			2 × 3 = 6
2 + 3 = 7			5 − 1 = 4
3 + 5 = 12			1 + 2 = 3
2 + 7 = 4			4 : 2 = 2
